# c-Abl, Lamellipodin, and Ena/VASP Proteins Cooperate in Dorsal Ruffling of Fibroblasts and Axonal Morphogenesis

**DOI:** 10.1016/j.cub.2010.03.048

**Published:** 2010-05-11

**Authors:** Magdalene Michael, Anne Vehlow, Christel Navarro, Matthias Krause

**Affiliations:** 1 Randall Division of Cell and Molecular Biophysics, King's College London, New Hunt's House, Guy's Campus, London SE1 1UL, UK

**Keywords:** CELLBIO

## Abstract

**Background:**

Tight regulation of cell motility is essential for many physiological processes, such as formation of a functional nervous system and wound healing. *Drosophila* Abl negatively regulates the actin cytoskeleton effector protein Ena during neuronal development in flies, and it has been postulated that this may occur through an unknown intermediary. Lamellipodin (Lpd) regulates cell motility and recruits Ena/VASP proteins (Ena, Mena, VASP, EVL) to the leading edge of cells. However, the regulation of this recruitment has remained unsolved.

**Results:**

Here we show that Lpd is a substrate of Abl kinases and binds to the Abl SH2 domain. Phosphorylation of Lpd positively regulates the interaction between Lpd and Ena/VASP proteins. Consistently, efficient recruitment of Mena and EVL to Lpd at the leading edge requires Abl kinases. Furthermore, transient Lpd phosphorylation by Abl kinases upon netrin-1 stimulation of primary cortical neurons positively correlates with an increase in Lpd-Mena coprecipitation. Lpd is also transiently phosphorylated by Abl kinases upon platelet-derived growth factor (PDGF) stimulation, regulates PDGF-induced dorsal ruffling of fibroblasts and axonal morphogenesis, and cooperates with c-Abl in an Ena/VASP-dependent manner.

**Conclusions:**

Our findings suggest that Abl kinases positively regulate Lpd-Ena/VASP interaction, Ena/VASP recruitment to Lpd at the leading edge, and Lpd-Ena/VASP function in axonal morphogenesis and in PDGF-induced dorsal ruffling. Our data do not support the suggested negative regulatory role of Abl for Ena. Instead, we propose that Lpd is the hitherto unknown intermediary between Abl and Ena/VASP proteins.

## Highlights

► Abl kinases transiently phosphorylate Lpd upon PDGF and netrin stimulation ► Phosphorylation of Lpd by c-Abl positively regulates Lpd-Ena/VASP interaction ► Lpd, Ena/VASP proteins, and Abl kinases regulate dorsal ruffling of fibroblasts ► c-Abl and Lpd promote axonal morphogenesis in an Ena/VASP-dependent manner

## Introduction

*Drosophila ena* was originally identified as a suppressor of lethality induced by mutations in *d-abl*, and it was postulated that *abl* and *ena* negatively regulate each other [1, 2]. Both the Abl tyrosine kinase family (D-Abl, vertebrate c-Abl and Arg) and the Ena/VASP family (Ena, vertebrate Mena, VASP, and EVL) act downstream of the netrin-1 axon guidance receptor DCC and regulate cell motility [3–6]. Abl kinases regulate platelet-derived growth factor (PDGF)-induced dorsal ruffling of fibroblasts, but it is not known whether Lamellipodin (Lpd) or Ena/VASP proteins function in this pathway [7].

Ena/VASP proteins play a crucial role in cell motility by antagonizing actin filament capping. This alters the geometry of the actin network toward longer, less-branched filaments, thereby changing the speed and persistence of lamellipodia [3, 5, 8]. Ena/VASP proteins are recruited to the leading edge through interactions between their EVH1 domain and FP4 motifs within Lpd, a member of the MRL family of Ras effector proteins, which includes *C. elegans* MIG-10, vertebrate RIAM and Lpd, and *Drosophila* Pico [9–13]. Lpd contains a proline-rich region harboring potential SH3-binding sites and a PH domain that mediates membrane targeting [9]. We have shown previously that Lpd is required for lamellipodia formation and that Lpd overexpression increases the speed of lamellipodial protrusion in an Ena/VASP-dependent manner [9].

Lpd-dependent recruitment of Ena/VASP proteins to the leading edge needs to be tightly regulated in order to precisely control lamellipodia formation, but it is not known how this is achieved. Studies in *Drosophila* have suggested that Abl regulates Ena localization [14]. Yet how the localization of Ena/VASP proteins is controlled, and whether Abl plays a role in this process in vertebrates, remains unclear.

Here we show that phosphorylation of Lpd by c-Abl increases its interaction with Ena/VASP proteins. Consistently, efficient recruitment of Mena and EVL to Lpd-positive lamellipodia requires Abl kinases. We provide evidence that Lpd and Ena/VASP proteins regulate dorsal ruffling of fibroblasts upon PDGF treatment and that Lpd function in this process is controlled by Abl kinases and mediated by Ena/VASP proteins. Furthermore, we demonstrate that both Lpd and c-Abl cooperate during axonal morphogenesis in an Ena/VASP-dependent manner. Our data do not support the suggested antagonistic roles of Abl and Ena, and we propose an alternative hypothesis that Abl kinases, via Lpd, positively regulate Ena/VASP proteins.

## Results

### Lamellipodin Is a Substrate for Abl Kinases

**Figure 1:**
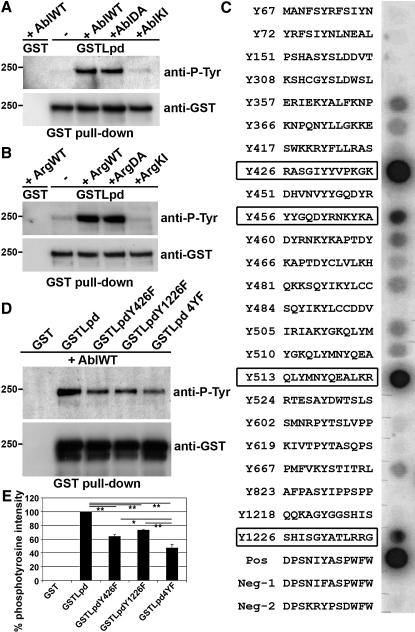
Lpd Is a Substrate of c-Abl and Arg Kinases (A and B) HEK293FT cells were transfected with GST-Lpd with wild-type AblWT (A) or ArgWT (B), dominant-active AblDA (A) or ArgDA (B), kinase-inactive c-Abl (AblKI) (A) or Arg (ArgKI) (B), or GST with AblWT or ArgWT (negative control). GST pull-down from lysates was followed by western blotting with anti-phosphotyrosine antibodies in (A), (B), and (D). (C) 24 peptides representing all tyrosine residues in Lpd, immobilized on a membrane, were incubated with c-Abl kinase and γ-^32^P-ATP and analyzed by autoradiography. (D and E) HEK293FT cells were transfected with GST-Lpd Y to F mutants and wild-type c-Abl (AblWT). (E) Quantification of blots by densitometry from three independent experiments. ^∗^p < 0.05; ^∗∗^p < 0.01 (one-way analysis of variance [ANOVA]). Error bars represent standard error of the mean (SEM). See also Figure S1.

Tyrosine kinases play an essential role in the propagation of signal transduction events [15]. Interestingly, phosphorylation site prediction software (Scansite) identified a putative c-Abl phosphorylation site in Lpd. To test this, we overexpressed GST-Lpd (Figure 1A) or HA-Lpd (see Figure S1A available online) with wild-type, dominant-active, or kinase-inactive c-Abl and found that Lpd is phosphorylated by wild-type and dominant-active, but not kinase-inactive, c-Abl. We could not detect any tyrosine phosphorylation of the Lpd-related protein RIAM in the presence of c-Abl (data not shown), suggesting that the posttranscriptional modification by Abl kinase is specific for Lpd in the vertebrate MRL family.

Cotransfection of GST-Lpd with the c-Abl-related kinase Arg or dominant-active but not kinase-inactive Arg also resulted in phosphorylation of GST-Lpd (Figure 1B), indicating that Lpd is a substrate of all members of the c-Abl family.

To identify the c-Abl phosphorylation sites in Lpd, we tested the ability of purified c-Abl to phosphorylate 24 immobilized peptides harboring all tyrosine residues within the Lpd amino acid sequence. This analysis revealed that, in vitro, four Lpd peptides harboring tyrosines (Y426, Y456, Y513, Y1226) are highly phosphorylated, and eight additional peptides are phosphorylated to a lesser extent (Figure 1C).

Three of the tyrosine residues were verified as c-Abl phosphorylation sites in full-length Lpd in cells, because nonphosphorylatable single point mutations located in the PH domain (Y426, Y456) or at the C terminus (Y1226) each reduced c-Abl phosphorylation of Lpd (Figures 1D and 1E; Figure S1B). However, we observed no reduction in phosphorylation of Y513 just behind the PH domain in the context of full-length Lpd (Figure S1B), indicating that Y513 might not be accessible for c-Abl phosphorylation in the folded protein under the condition tested. Nevertheless, mutation of all four tyrosines (GST-Lpd4YF) further reduced Lpd phosphorylation (Figures 1D and 1E), suggesting that Lpd is a novel Abl family substrate harboring three major c-Abl phosphorylation sites.

### Endogenous Lpd Is Phosphorylated by c-Abl upon PDGF Stimulation

**Figure 2:**
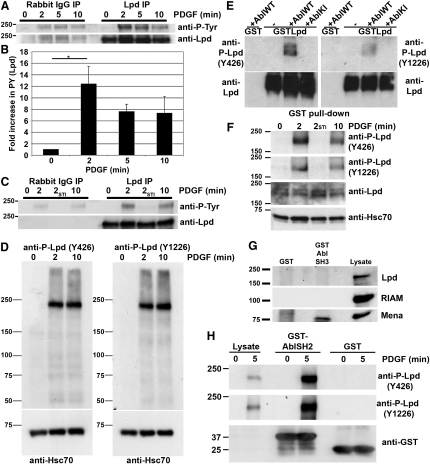
Endogenous Lpd Is Phosphorylated at Y426 and Y1226 by c-Abl upon Platelet-Derived Growth Factor Stimulation (A) Serum-starved NIH 3T3 cells were stimulated with 50 ng/ml platelet-derived growth factor (PDGF). Lpd was immunoprecipitated and immunoblotted with anti-phosphotyrosine antibodies. (B) Densitometry: control values were subtracted from values of phosphotyrosine bands in (A) (three independent experiments) and represented as fold increase compared to 0 min. ^∗^p < 0.05 (Student's t test). Error bars represent SEM. (C) Serum-starved NIH 3T3 cells were treated with dimethyl sulfoxide (DMSO) or 1 μM STI-571 for 2 hr prior to stimulation with PDGF. Lysates were immunoblotted with anti-phosphotyrosine antibodies. (D) Serum-starved NIH 3T3 cells were stimulated with 50 ng/ml PDGF and cell lysates were immunoblotted with Y426 and Y1226 phosphospecific Lpd antibodies and anti-Hsc70 as loading control. (E) HEK293FT cells were transfected with GST-Lpd, wild-type (AblWT), or kinase-inactive (AblKI) c-Abl. GST pull-down from lysates was followed by immunoblotting with Y426 and Y1226 phosphospecific Lpd antibodies. (F) Serum-starved NIH 3T3 cells were treated with DMSO or 1 μM STI-571 for 2 hr and stimulated with PDGF, and lysates were immunoblotted with phosphospecific Lpd antibodies. (G) GST-Abl-SH3-agarose was incubated with lysates of NIH 3T3 cells and bound Lpd, RIAM, or Mena detected in a western blot. GST-agarose was used as a negative control. (H) Lpd was pulled down from lysates of PDGF-stimulated NIH 3T3 cells with GST-Abl-SH2-agarose and was immunoblotted with anti-phospho-Lpd antibodies. GST-agarose was used as a negative control. (A–H) All experiments have been repeated at least three times. See also Figure S2.

Because Abl kinases are activated upon PDGF receptor ligation [6], we investigated whether endogenous Lpd is phosphorylated downstream of the PDGF receptor. We stimulated serum-starved NIH 3T3 cells with PDGF for various time points and immunoprecipitated endogenous Lpd from cell lysates. Lpd phosphorylation peaked within 2 min of PDGF stimulation and subsequently declined (Figures 2A and 2B).

To further investigate the phosphorylation of endogenous Lpd, we generated phosphospecific antibodies against two of the major phosphorylation sites, in the PH domain (anti-P-Lpd-Y426) and in the C-terminal region (anti-P-Lpd-Y1226), respectively. These antibodies detected phosphorylated Lpd only when Lpd was cotransfected with wild-type but not with kinase-inactive c-Abl (Figure 2E), or they detected endogenous phosphorylated Lpd on western blots of PDGF-stimulated NIH 3T3 cell lysates (Figure 2D).

To explore whether Abl kinases are required for the phosphorylation of Lpd upon PDGF stimulation, we pretreated NIH 3T3 cells with a low concentration (1 μM) of the Abl kinase inhibitor STI571 before stimulating the cells with PDGF for 2 min. Tyrosine phosphorylation of total Lpd (Figure 2C) and at Lpd residues Y426 and Y1226 (Figure 2F) was abrogated by Abl inhibitor treatment, suggesting that endogenous Lpd is transiently phosphorylated by Abl kinases upon PDGF stimulation.

c-Abl can be activated by interaction with its substrates [16], and we observed that wild-type c-Abl, as well as dominant-active c-Abl (lacking the SH3 domain) and kinase-inactive c-Abl, coprecipitated with GST-Lpd (Figure S2A). However, we did not observe an increase in c-Abl kinase activity upon cotransfection with Lpd in in vitro kinase assays (Figure S2B), indicating that Lpd phosphorylation requires activation of c-Abl by upstream signals such as PDGF receptor activation. To test whether binding is mediated by the SH3 or SH2 domain of c-Abl, we performed pull-down assays from NIH 3T3 cell lysates with purified GST-Abl-SH3 or SH2 domains. As a positive control, we found that the Abl-SH3 domain pulled down Mena as previously published [17]. In contrast, Lpd and RIAM did not interact with the Abl SH3 domain (Figure 2G). However, Lpd phosphorylated on both Y426 and Y1226 precipitated with the GST-Abl-SH2 domain from lysates of PDGF-stimulated NIH 3T3 cells (Figure 2H), suggesting that a complex between Lpd and c-Abl is formed upon growth factor stimulation and that complex formation is mediated via the Abl-SH2 domain.

### Phosphorylation of Lpd by c-Abl Positively Regulates Lpd-Ena/VASP Interaction

**Figure 3:**
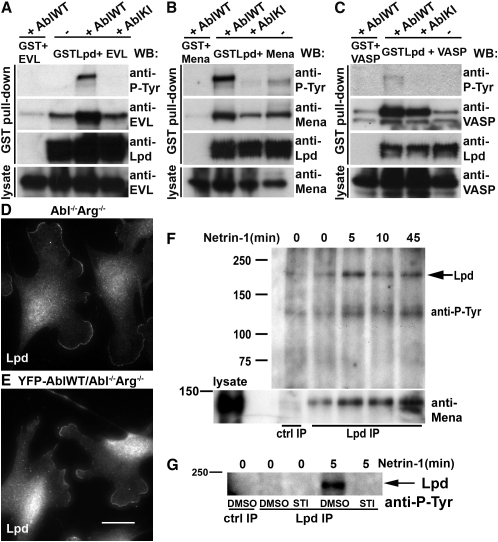
Lpd Interaction with Ena/VASP Proteins Is Regulated by c-Abl (A–C) HEK293FT cells were transfected with GST-Lpd, EVL, Mena, or VASP and AblWT or AblKI. GST pull-down on lysates was followed by western blotting with anti-phosphotyrosine, Lpd, and EVL (A), Mena (B), or VASP (C) antibodies. (D and E) Abl^−/−^Arg^−/−^ and YFP-AblWT/Abl^−/−^Arg^−/−^ cells were fixed and stained with Lpd antibodies. Scale bar represents 30 μm. (F and G) Primary cortical neurons were cultured for 36 hr without (F) or with (G) 10 μM STI571 or DMSO, were stimulated with 300 ng/ml netrin-1, and were lysed. Lpd was immunoprecipitated and examined for tyrosine phosphorylation (F and G) and Mena coprecipitation (F) by western blotting. Purified rabbit IgG was used for the control immunoprecipitation. (A–G) All experiments have been repeated at least three times. See also Figure S3.

A major function of Lpd is to recruit Ena/VASP proteins to the leading edge, an event that needs to be tightly regulated for controlled lamellipodia formation [9]. In agreement with a potential regulation of Lpd by c-Abl, we observed that Lpd colocalized with endogenous c-Abl in clusters at the very edge of lamellipodia in NIH 3T3 fibroblasts (Figure S3A) and growth cones of primary hippocampal neurons (Figure S3B). To test whether Lpd localization at the leading edge might be regulated by Abl kinases, we used mouse embryonic fibroblasts lacking both c-Abl and Arg [18] and rescued this cell line with YFP-tagged wild-type c-Abl (Figure S3C). Interestingly, Lpd localized to lamellipodia in Abl^−/−^Arg^−/−^ fibroblasts (Figure 3D), as well as in c-Abl-expressing cells (Figure 3E), indicating that Lpd localization is not regulated by Abl kinases.

To explore the possibility that phosphorylation of Lpd by c-Abl may regulate Lpd-Ena/VASP interaction, we cotransfected GST-Lpd with wild-type or kinase-inactive c-Abl and tagged EVL, Mena, or VASP. Pull-down of GST-Lpd and western blot for Ena/VASP proteins revealed that cotransfection of wild-type c-Abl increased the interaction between Lpd and EVL or between Lpd and Mena (Figures 3A and 3B). Consistently, cotransfection with kinase-inactive c-Abl decreased the interaction between Lpd and Mena (Figure 3B). Interestingly, Lpd was less phosphorylated by c-Abl when VASP was coexpressed. VASP coprecipitated with Lpd regardless of whether kinase-inactive or wild-type c-Abl was expressed (Figure 3C). However, cotransfection of wild-type c-Abl increased the interaction between VASP and Lpd compared to kinase-inactive c-Abl (Figure 3C). Taken together, this indicates that c-Abl positively regulates the interaction between Lpd and Ena/VASP proteins.

### Lpd Is Phosphorylated upon Netrin-1 Stimulation, and This Positively Correlates with Increased Mena Coprecipitation

The Lpd and Ena/VASP *C. elegans* orthologs *mig-10* and *unc-34* function downstream of the netrin receptor *unc-40*/DCC [4]. To explore whether netrin-1 stimulation regulates endogenous Lpd-Mena interaction in vertebrates, we stimulated primary cortical neurons with the axon guidance cue netrin-1 and observed a transient increase in tyrosine phosphorylation of Lpd within 5 min (Figure 3F). Interestingly, the amount of Mena coprecipitating with Lpd also increased after 5 min of netrin-1 stimulation (Figure 3F). To test whether Lpd phosphorylation upon netrin-1 treatment is mediated by Abl kinases, we stimulated STI571-treated cortical neurons with netrin-1 and observed a loss of Lpd phosphorylation upon Abl kinase inhibition (Figure 3G). Taken together, this suggests that netrin/DCC signaling induces Abl kinase-dependent transient phosphorylation of Lpd to increase Mena recruitment.

### Leading-Edge Localization of Ena/VASP Proteins Is Differentially Regulated by Abl Kinases

**Figure 4:**
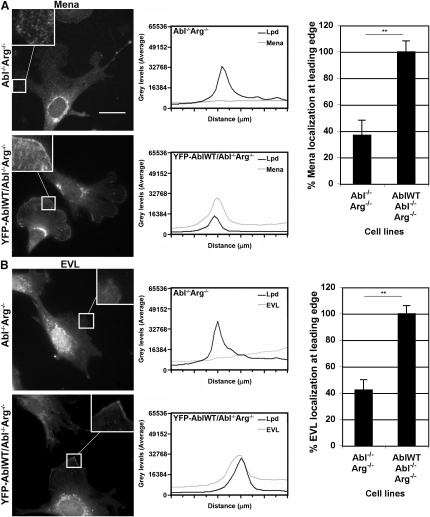
Abl Kinases Regulate Leading-Edge Localization of Mena and EVL (A and B) Abl^−/−^Arg^−/−^ and YFP-AblWT/Abl^−/−^Arg^−/−^ cells were plated, fixed, and stained with Lpd (Figure S4C) and Mena (A) or EVL (B) antibodies. Leading-edge localization of Mena and EVL was quantified via line scans in cells with Lpd leading-edge staining. Per cell type, n = 60 cells were analyzed from three independent experiments. Values above the median were used to classify positive leading-edge localization. The number of cells with positive leading-edge localization in Abl^−/−^Arg^−/−^ cells was represented as a percentage in comparison to YFP-AblWT/Abl^−/−^Arg^−/−^ cells. ^∗∗^p < 0.01 (Student's t test). Error bars represent SEM. See also Figure S4.

c-Abl-dependent phosphorylation of Lpd positively regulates its interaction with Ena/VASP proteins (Figure 3). Therefore, we investigated whether localization of Ena/VASP proteins to the leading edge is regulated by Abl kinases. Abl^−/−^Arg^−/−^ and YFP-c-Abl/Abl^−/−^Arg^−/−^ fibroblasts express Mena, VASP, and EVL (Figure S4A). VASP was recruited to the leading edge regardless of the presence of Abl kinases (Figure S4B). Surprisingly, Mena and EVL were not efficiently recruited to Lpd-positive lamellipodia in Abl^−/−^Arg^−/−^ fibroblasts but localized to the leading edge in c-Abl-expressing Abl^−/−^Arg^−/−^ fibroblasts (Figures 4A and 4B; Figure S4C). This indicates that Mena and EVL, but not VASP recruitment to lamellipodia, depend on c-Abl, and this may be mediated by phosphorylation of Lpd.

### Lpd and Ena/VASP Proteins Regulate PDGF-Induced Dorsal Ruffles

**Figure 5:**
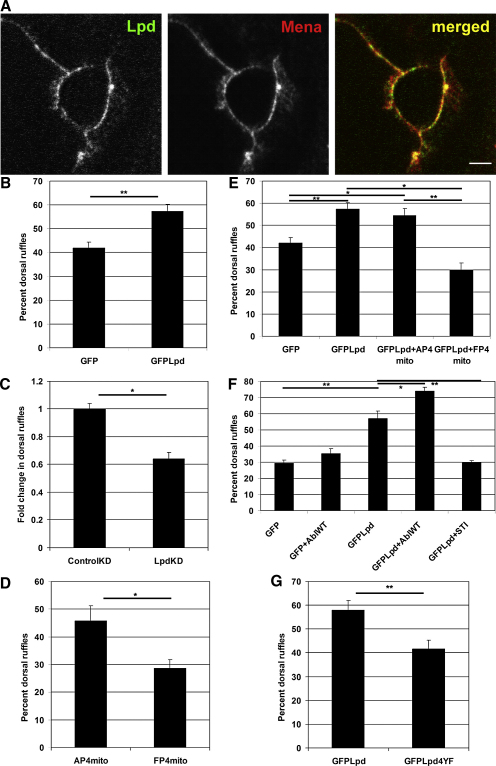
Lpd Regulates the PDGF-Induced Dorsal Ruffle Response in an Ena/VASP-Dependent Manner (A) Single confocal plane at the dorsal surface of PDGF (5 min, 30 ng/ml)-stimulated NIH 3T3 cells stained with Lpd and Mena antibodies. Scale bar represents 2 μm. (B–G) NIH 3T3 cells were stimulated with 30 ng/ml PDGF for 5 min and fixed, and F-actin was stained with A568-phalloidin. Coverslips were automatically scanned, and transfected cells with dorsal ruffles were quantified. (B) GFP-Lpd overexpression increases the dorsal ruffle response. (C) Lpd knockdown was achieved by transfection of a plasmid with an Lpd small hairpin RNA or scrambled control and puromycin resistance. After selection, cells were serum starved and stimulated with 30 ng/ml PDGF. (D) NIH 3T3 cells were transfected with mRFP1-FP4-mito to delocalize all Ena/VASP proteins or mRFP1-AP4-mito as control. (E) NIH 3T3 cells were transfected with GFP, GFP-Lpd alone, or in combination with mRFP1-FP4-mito or mRFP1-AP4-mito as control. (F) NIH 3T3 cells were transfected with GFP or GFP-Lpd alone, or in combination with wild-type c-Abl (AblWT) or were pretreated for 2 hr with 1 μM STI571. (G) NIH 3T3 cells were transfected with GFP, GFP-Lpd, or GFP-Lpd4YF (mutated at Y426, Y456, Y513, Y1226). ^∗^p < 0.05; ^∗∗^p < 0.01 (B, C, D, and G: Student's t test; E and F: one-way ANOVA). Error bars represent SEM.

Abl kinases regulate dorsal ruffling of fibroblasts upon PDGF stimulation [7]. We observed that Lpd localizes to the rim of PDGF-induced dorsal ruffles of NIH 3T3 fibroblasts where it colocalizes with Mena (Figure 5A). Lpd overexpression significantly increased dorsal ruffling upon PDGF treatment (Figure 5B). Conversely, Lpd knockdown decreased dorsal ruffling (Figure 5C), suggesting that Lpd regulates this response.

To test the role of Ena/VASP for dorsal ruffle formation, we employed a well-established strategy to artificially delocalize all Ena/VASP proteins [19, 20]. This strategy relies on the specific interaction of the EVH1 domain of Ena/VASP proteins with a proline-rich motif (D/EFPPPPXD/ED/E) [5]. Expressing four EVH1-binding sites fused to a mitochondrial membrane anchor (FP4-mito) delocalizes all Ena/VASP proteins to mitochondria, thereby rendering them nonfunctional. Mutation of phenylalanine to alanine in the FP4 motif abolishes binding to Ena/VASP proteins (AP4-mito, negative control) [19]. Delocalization of all Ena/VASP proteins in NIH 3T3 fibroblasts significantly reduced dorsal ruffling (Figure 5D), suggesting that Ena/VASP function is important for this process.

Because Lpd might regulate dorsal ruffling by recruiting Ena/VASP proteins, we tested whether the increase in dorsal ruffling induced by Lpd overexpression was Ena/VASP dependent. Coexpressing AP4-mito with GFP-Lpd was as efficient as GFP-Lpd alone in increasing dorsal ruffling (Figure 5E). However, coexpressing GFP-Lpd with FP4-mito completely abolished this effect (Figure 5E), indicating that Ena/VASP proteins mediate Lpd-induced dorsal ruffling.

### Lpd Function during Dorsal Ruffling Is Regulated by c-Abl

Because c-Abl [7] and Lpd (Figure 5C) positively affect dorsal ruffling, we investigated whether c-Abl might regulate Lpd in this process. Coexpression of GFP-Lpd with c-Abl further increased the GFP-Lpd overexpression phenotype (Figure 5F), indicating that c-Abl and Lpd cooperate in this process. To test whether c-Abl kinase activity is required for Lpd function, we pretreated GFP-Lpd-expressing fibroblasts with the Abl inhibitor STI571. This treatment abolished the GFP-Lpd-induced increase in dorsal ruffling (Figure 5F), indicating that Lpd function requires phosphorylation by Abl kinases. In contrast to GFP-Lpd, overexpression of GFP-Lpd4YF (Figure 1D) did not increase the dorsal ruffling response (Figure 5G), suggesting that phosphorylation of Lpd at the identified c-Abl sites is required for its function in dorsal ruffling.

### Lpd Regulates Axonal Morphogenesis

**Figure 6:**
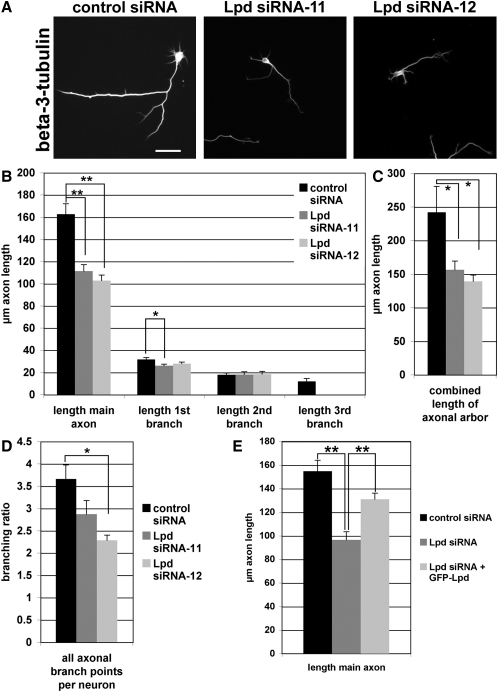
Lpd Is Required for Axonal Morphogenesis (A) Hippocampal neurons transfected with Lpd small interfering RNAs (siRNAs) or control siRNA plated on laminin-coated coverslips were stained with anti-beta(III)-tubulin and Tau-1 antibodies (Figure S5B). Scale bar represents 50 μm. (B) Axons were identified with Tau-1 staining (Figure S5B), and length of main axon and branches were manually quantified with NeuronJ from anti-beta(III)-tubulin stainings. Three independent experiments were performed: control siRNA, n = 128 neurons; Lpd siRNA-11, n = 174 neurons; Lpd siRNA-12, n = 184 neurons. (C and D) The total length of all axonal branches per neuron (C) and the number of branch points per axon (D) were calculated. (E) Axonal length of primary hippocampal neurons cotransfected with either control siRNA and GFP, Lpd siRNA and GFP, or Lpd siRNA and human GFP-Lpd was quantified. Three independent experiments were performed: control siRNA + EGFP, n = 102 neurons; Lpd siRNA-11+EGFP, n = 92 neurons; Lpd siRNA-11+human Lpd-EGFP, n = 89 neurons. ^∗^p < 0.05; ^∗∗^p < 0.001 (one-way ANOVA). Error bars represent SEM. See also Figure S5.

Abl kinases regulate neuronal morphogenesis and are required for the integrin-dependent, laminin-induced increase in axonal length and branching [6]. To explore whether Lpd is required for neuronal morphogenesis, we transfected primary hippocampal neurons with two Lpd small interfering RNAs (siRNAs) that are able to efficiently knock down Lpd or a control siRNA (Figure S5A). Axons were detected by Tau-1 staining (Figure S5B). The length, branching, and number of primary dendrites per neuron were not significantly altered upon Lpd knockdown (data not shown). Control siRNA-transfected neurons formed long, highly branched axons when plated on laminin for 2 days (Figure 6A). In contrast, the length of the main axon was significantly shorter (reduced by 30%) in neurons transfected with Lpd-specific siRNAs (Figures 6A and 6B). The sum of the length of the main axon and all axonal branches was further decreased (Figure 6C). Consistently, axonal branching was highly impaired in the Lpd siRNA-transfected neurons (Figure 6D). Coexpression of human GFP-Lpd together with the mouse Lpd siRNA rescued the Lpd knockdown phenotype, verifying that the observed phenotype was not due to an off-target effect (Figure 6E). Taken together, these results suggest that Lpd regulates elongation and branching of axons.

### c-Abl Cooperates with Lpd in an Ena/VASP-Dependent Manner in the Regulation of Axonal Morphogenesis

**Figure 7:**
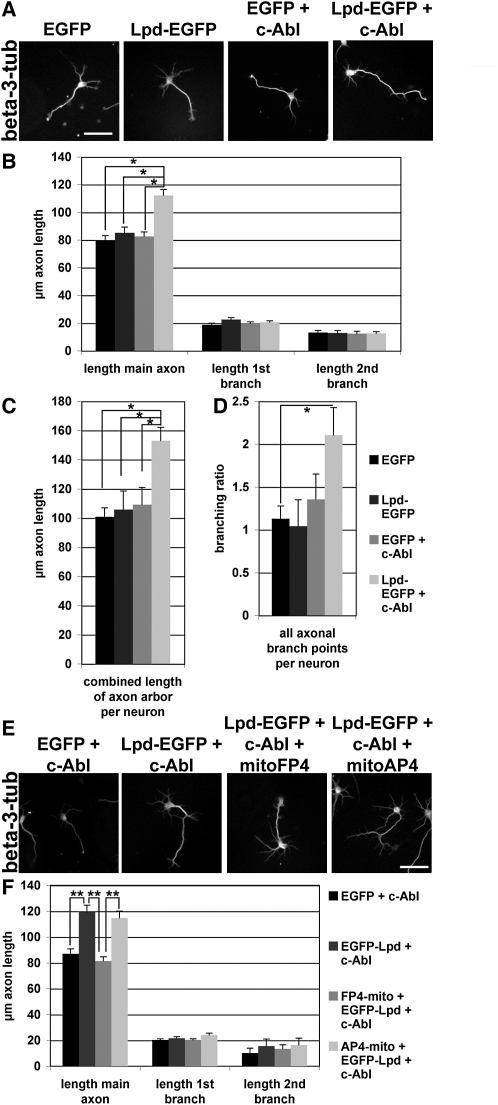
Lpd Cooperates with c-Abl in an Ena/VASP-Dependent Manner in the Regulation of Axonal Morphogenesis (A–D) Hippocampal neurons transfected with EGFP control, Lpd-EGFP, wild-type YFP-c-Abl+EGFP control, or Lpd-EGFP + wild-type YFP-c-Abl plated on poly-D-lysine-coated coverslips were stained with anti-beta(III)-tubulin (A) and Tau-1 (Figure S5C) antibodies. Scale bars in (A) and (E) represent 50 μm. (B and F) Axons were identified with Tau-1 staining (Figure S5C), and length of main axon and branches were manually quantified with NeuronJ from anti-beta(III)-tubulin stainings. Three independent experiments were performed: control EGFP, n = 137 neurons; Lpd-EGFP, n = 134 neurons; EGFP+c-Abl, n = 161 neurons; Lpd-EGFP+c-Abl, n = 133 neurons. (C and D) The total length of all axonal branches per neuron (C) and the number of branch points per axon (D) were calculated. (E and F) Hippocampal neurons transfected with wild-type YFP-c-Abl+EGFP control, Lpd-EGFP + wild-type YFP-c-Abl with mRFP1-FP4-mito, or AP4-mito and plated onto poly-D-lysine-coated coverslips were stained with anti-beta(III)-tubulin and Tau-1 antibodies (Figure S5D). (F) Data from three independent experiments: EGFP+c-Abl, n = 140 neurons; Lpd-EGFP+c-Abl, n = 94 neurons; Lpd-EGFP+c-Abl+FP4-mito, n = 121 neurons; Lpd-EGFP+c-Abl+AP4-mito, n = 90 neurons. ^∗^p < 0.05; ^∗∗^p < 0.001 (one-way ANOVA). Error bars represent SEM.

In primary neurons, overexpression of dominant-active c-Abl, but not wild-type c-Abl, resulted in increased neurite growth and branching, because wild-type c-Abl is tightly autoinhibited [21, 22]. Because Lpd is positively regulated by c-Abl and contributes to axonal extension and branching, we explored the possibility that Lpd and c-Abl may cooperate to regulate this process. We transfected primary hippocampal neurons either with empty EGFP vector, EGFP-Lpd, or wild-type YFP-c-Abl and plated neurons after transfection onto poly-D-lysine-coated coverslips, an adhesive substratum that does not activate integrins. This induces only limited neurite outgrowth and branching when compared to neurons plated on laminin, thereby allowing us to investigate potential increases in length and branching induced by overexpression. Overexpression of wild-type c-Abl or EGFP-Lpd alone had no effect on length and branching of either axons and dendrites (Figures 7A–7D; data not shown). In contrast, co-overexpression of Lpd with wild-type c-Abl induced a significant increase in length of the main axon (Figures 7A and 7B). The sum of the length of the main axon and all axonal branches was further increased (Figure 7C). Consistently, branching was doubled in the neurons transfected with Lpd and c-Abl (Figures 7A and 7D), indicating that c-Abl and Lpd cooperate to positively regulate axonal length and branching.

To explore whether cooperation of c-Abl with Lpd in axonal morphogenesis is mediated by Ena/VASP proteins, we co-overexpressed c-Abl and Lpd with or without FP4-mito or AP4-mito constructs to delocalize all Ena/VASP proteins. Co-overexpression of c-Abl and EGFP-Lpd or of c-Abl, EGFP-Lpd, and AP4-mito significantly increased the length of the main axon, compared to c-Abl with EGFP (Figures 7E and 7F). Importantly, co-overexpression of c-Abl, EGFP-Lpd, and FP4-mito failed to induce an increase in the length of the main axon (Figures 7E and 7F), suggesting that axonal elongation induced by cooperation of c-Abl and Lpd is mediated by Ena/VASP proteins.

## Discussion

In this study we reveal Lpd as a novel substrate of Abl kinases that is transiently phosphorylated upon PDGF and netrin-1 stimulation. We found that Lpd is phosphorylated by c-Abl at its PH domain and at the C terminus. Phosphorylation by Abl kinases at the PH domain does not regulate the interaction of Lpd with the plasma membrane because Lpd localizes at lamellipodia in Abl^−/−^Arg^−/−^ fibroblasts (Figure 3).

We observed that the interaction between Lpd and Ena/VASP proteins is positively regulated by c-Abl. However, Ena/VASP-binding sites in Lpd are not directly phosphorylated by Abl kinases. The phosphorylation of Lpd might alter its tertiary structure, thereby unmasking the Ena/VASP-binding sites. Furthermore, we noticed differences in the biochemical behavior of individual Ena/VASP proteins. We found that wild-type, dominant-active, and kinase-inactive c-Abl can coprecipitate with Lpd (Figure S2A). Wild-type and dominant-active, but not kinase-inactive, c-Abl may bind directly to phosphorylated Lpd via the c-Abl SH2 domain. In contrast, kinase-inactive c-Abl may bind indirectly to Lpd via Ena/VASP, because Ena/VASP proteins can directly interact with the c-Abl SH3 domain [5] and Ena/VASP can interact with FP4 motifs in Lpd [9]. Furthermore, VASP localization to the leading edge is independent of Abl kinases (Figure S4B), suggesting that its recruitment to this site can also occur without Lpd phosphorylation by Abl kinases. In addition, only Lpd-Mena, and not Lpd-EVL or Lpd-VASP interaction, is inhibited by expression of kinase-inactive c-Abl, suggesting that c-Abl may also regulate Mena directly. This might be achieved by phosphorylation of Ena or Mena, because they are substrates of Abl [5]. We believe that this is unlikely because we did not observe any tyrosine phosphorylation of Mena when we co-overexpressed GST-Lpd, Mena, and c-Abl (data not shown; Figure 3). Furthermore, direct phosphorylation of Ena by D-Abl is not required for regulation of Ena function because expression of a nonphosphorylatable Ena mutant rescued axon guidance defects in the *Drosophila ena* mutant [5, 23]. However, Abl kinase activity is required for its function in axon guidance [24, 25], suggesting that D-Abl must phosphorylate some other component. It was postulated that Abl regulates Ena's location in cells because Ena is mislocalized in *d-abl* mutant flies [14]. Taken together, this suggests that Abl regulates Ena localization indirectly by phosphorylating an unknown protein. In this study we have discovered that the interaction between Lpd and Ena/VASP proteins is positively regulated by c-Abl. Therefore, we suggest that Lpd is this hitherto unknown intermediary between Abl and Ena/VASP and that the differential formation of trimolecular complexes between Lpd, c-Abl, and individual Ena/VASP proteins allows for fine tuning of signaling responses.

This positive regulation of Lpd and Ena/VASP by Abl is surprising because it was postulated that *Drosophila d-abl* negatively regulates *ena* function [1, 2]. How can both be reconciled? Unexpectedly, comparing known cellular functions of Abl kinases and Ena/VASP proteins revealed that many functions are very similar and not in opposition to each other. First, overexpression of both Abl kinases and Ena/VASP proteins increases filopodia formation [20, 22, 26]. Second, analysis of knockout fibroblasts lacking Ena/VASP proteins or Abl kinases revealed that they migrated faster compared to cells re-expressing physiological levels of the respective proteins [8, 19, 27, 28]. This indicates that both Abl kinases and Ena/VASP proteins negatively regulate whole-cell migration [8, 19, 27, 29, 30]. Third, both Abl/Arg or Ena/VASP knockout mice have neurulation defects during development and die of hemorrhage [18, 31–34]. Finally, both *Drosophila ena* and *d-abl* mutants have defects in longitudinal and commissural axon tracts [2, 4, 6].

How can mutations in *ena* ameliorate *abl* phenotypes when Abl is a positive regulator of Ena? We suggest that Abl positively regulates the correct subcellular localization of Ena/VASP proteins, which is consistent with our observations and with those in *d-abl* mutant flies [14]. When D-Abl is absent, Ena accumulates ectopically where excess F-actin is observed [14]. Consistently, reduction of *ena* in *abl* mutant flies restores viability [1, 2]. Therefore, rescue of viability can be equally well explained by a positive regulation of Ena by Abl.

Lpd is transiently phosphorylated upon stimulation of primary cortical neurons with netrin-1 and is accompanied by an increased Lpd-Mena interaction. Treatment of neurons with netrin-1 causes increased formation of lamellipodia within 5 to 10 min [20], which correlates well with the time course of Lpd phosphorylation and increased interaction with Mena. In *C. elegans*, *mig-10* and *unc-34/ena* function together to mediate *unc-6*/netrin-dependent axon guidance decisions [4]. Our data indicate that cooperation between Lpd and Ena/VASP proteins downstream of the netrin receptor also occurs in vertebrates.

We observed a transient phosphorylation of Lpd upon PDGF receptor stimulation and found that Lpd and Ena/VASP proteins contribute to the PDGF-induced dorsal ruffle response of fibroblasts. Our data indicate that Lpd function in the dorsal ruffle response is regulated by Abl kinases and mediated by Ena/VASP proteins. In lamellipodia, Ena/VASP proteins increase elongation of actin filaments by antagonizing actin filament capping [8]. N-WASP and the Arp2/3 complex facilitate actin nucleation off of the side of existing actin filaments, and cortactin stabilizes these actin branches [35]. N-WASP [6], cortactin [36], and Lpd (this study) are positively regulated by Abl kinases and function to regulate dorsal ruffling. A fine balance of Arp2/3-mediated branching and Ena/VASP-mediated elongation of actin filaments regulates the actin ultrastructure during lamellipodia protrusion [8] and might also regulate dorsal ruffle formation. Therefore, positive regulation by Abl kinases and cooperation between Lpd, Ena/VASP, N-WASP, Arp2/3, and cortactin might be a more general mechanism to modulate the actin ultrastructure for different types of membrane protrusions.

In conclusion, we have identified Lpd as a component of PDGF and netrin-1 signaling pathways. Lpd is a substrate of Abl kinases, and c-Abl cooperates with Lpd in an Ena/VASP-dependent manner during the PDGF-induced dorsal ruffle response and axonal morphogenesis. Lpd's interaction with Ena/VASP proteins is positively regulated by Abl kinases. We propose that Lpd is the hitherto unknown intermediary between Abl and Ena/VASP. Our data do not support the suggested negative regulatory role of Abl for Ena, and we propose an alternative hypothesis that Abl kinases, via Lpd, positively regulate Ena/VASP proteins.

## Experimental Procedures

### Molecular Biology, Plasmids, and Reagents

See Supplemental Experimental Procedures.

### Cell Culture

NIH 3T3 (ATCC), HEK293FT (Invitrogen), and Abl^−/−^Arg^−/−^ MEFs (a kind gift from T. Koleske, Yale) were cultured in Dulbecco's modified Eagle's medium, penicillin, streptomycin, 10% fetal bovine serum, or calf serum (NIH 3T3) and transfected with Lipofectamine 2000 (Invitrogen). NIH 3T3 cells were serum starved (18 hr) before stimulation with 30 or 50 ng/ml PDGF. pK1-YFP-c-Abl and pCL-Eco were cotransfected in HEK293FT to generate retroviruses to transduce Abl^−/−^Arg^−/−^ MEFs, and YFP-positive cells were selected by fluorescence-activated cell sorting. See Supplemental Experimental Procedures for immunofluorescence analysis and imaging.

### Immunoprecipitations and Western Blotting

Primary cortical neurons were cultured for 36 hr before incubation with 300 ng/ml chicken netrin-1 (R&D Systems). All cells were lysed in glutathione S-transferase (GST) buffer (50 mM Tris-HCL, pH 7.4, 200 mM NaCl, 1% NP-40, 2 mM MgCl_2_, 10% glycerol, NaF + Na_3_VO_4_, complete mini tablets without EDTA, Roche). Precleared lysate was incubated with glutathione beads (Amersham) or with primary antibody or control IgG followed by protein A beads (Pierce) and was washed with GST buffer. Western blotting was performed as described [9].

### Peptide Array Assay

Custom-made immobilized peptide arrays (CR-UK) were incubated overnight at room temperature in kinase buffer (50 mM Tris-Hcl, 10 mM MgCl_2_, 1 mM EGTA, 2 mM DTT, 0.01% Brij 35) with 0.2 mg/ml bovine serum albumin (BSA) and 10 mM NaCl, were blocked for 45 min at 30°C in kinase buffer with 1 mg/ml BSA and 100 mM NaCl, and were incubated with kinase buffer + 0.2 mg/ml BSA + 120 units Abl kinase (NEB) + 24 μCi γ-^32^P-ATP for 2 hr at 30°C. Washed membranes (10 × 15 min 1 M NaCl, 3 × 5 min H_2_O, 3 × 5 min H_3_P0_4_, 3 × 5 min H_2_O, 2 × 2 min ethanol) were dried and analyzed with a Phosphorimager Typhoon 9200 (Amersham).

## Supplemental Information

Document S1. Supplemental Experimental Procedures and Five Figures
