# Supplementary material for: c-Abl, Lamellipodin, and Ena/VASP Proteins Cooperate in Dorsal Ruffling of Fibroblasts and Axonal Morphogenesis
Source: Curr Biol. 2010 May 11;20(9-9):783–91. doi: 10.1016/j.cub.2010.03.048 (PMC2946563; doi:10.1016/j.cub.2010.03.048)
Supplement: Document S1. Supplemental Experimental Procedures and Five Figures [file mmc1.pdf]

Current Biology, Volume *20*

Supplemental Information

c-Abl, Lamellipodin, and Ena/VASP Proteins Cooperate in Dorsal Ruffling of Fibroblasts and Axonal Morphogenesis

Magdalene Michael, Anne Vehlow, Christel Navarro, and Matthias Krause

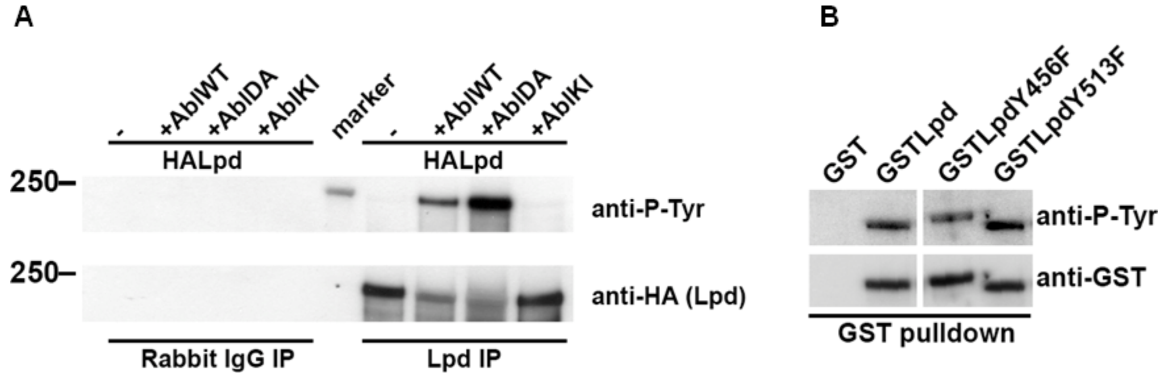

Figure S1.
(A) HA-tagged Lpd is phosphorylated by c-Abl. HEK293FT cells were transfected with HA-Lpd with and without wild type c-Abl (AblWT), dominant-active c-Abl (AblDA), or kinase-inactive c-Abl (AblKI). Immunoprecipitation was performed with Lpd specific rabbit polyclonal antibodies or purified rabbit IgG as control followed by Western blotting with anti-phosphotyrosine or anti-HA antibodies.
(B) Analysis of phosphorylation of GST-Lpd with mutations in the mapped c-Abl phosphorylation sites by c-Abl. HEK293FT cells were transfected with GST-Lpd Y to F mutants and wild type c-Abl (AblWT). GST pulldown was performed on lysates followed by Western blotting with anti-phosphotyrosine antibodies to assess phosphorylation of Lpd mutants.

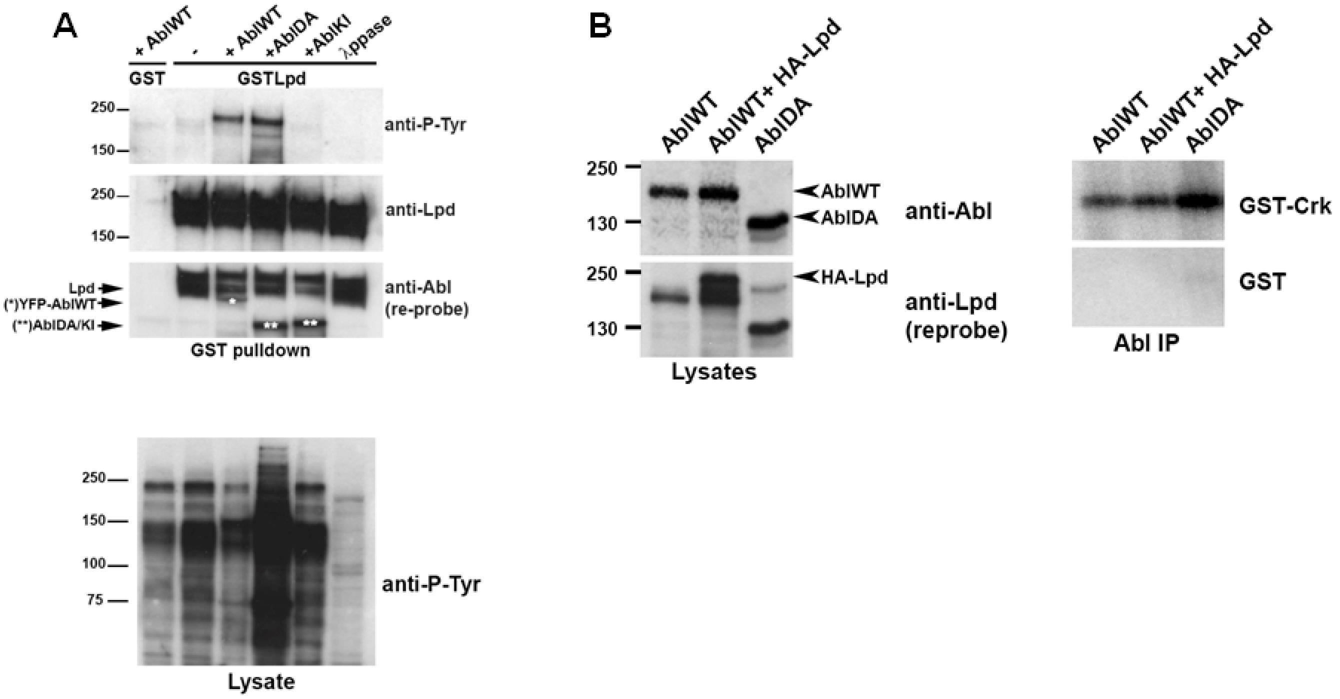

Figure S2.

(A) Lpd coprecipitates with c-Abl. HEK293FT cells were transfected with GST-Lpd with and without wild type c-Abl (AblWT), dominant-active c-Abl (AblDA), or kinase-inactive c-Abl (AblKI). In parallel, lysates of GST-Lpd transfected cells were treated *in vitro* with lambda phosphatase. Co-transfection of GST with wild type c-Abl served as a negative control. GST pulldowns were performed on the lysates followed by Western blotting with anti-phosphotyrosine or anti-c-Abl antibodies. Below: a parallel Western Blot of the cell lysates was probed with anti-phospho- tyrosine antibodies. Note that expression of wild type Abl does not increase overall tyrosine phosphorylation (compare lane two and three).

(B) Lpd does not activate c-Abl. HEK293FT cells were transfected with HA-Lpd with and without wild type c-Abl (AblWT), or dominant-active c-Abl (AblDA) alone. c-Abl immunoprecipitates were incubated with purified GST or GST-Crk on glutathione sepharose beads in the presence of γ-P^{32}-ATP. After extensive washes, beads were boiled with sample buffer and proteins resolved by SDS-PAGE. Phosphorylated GST-Crk in SDS-PAGE gel was detected using a phosphor-imager. Note that dominant active c-Abl can phosphorylate GST-Crk to a higher extend than wild type c-Abl and serves as a positive control.

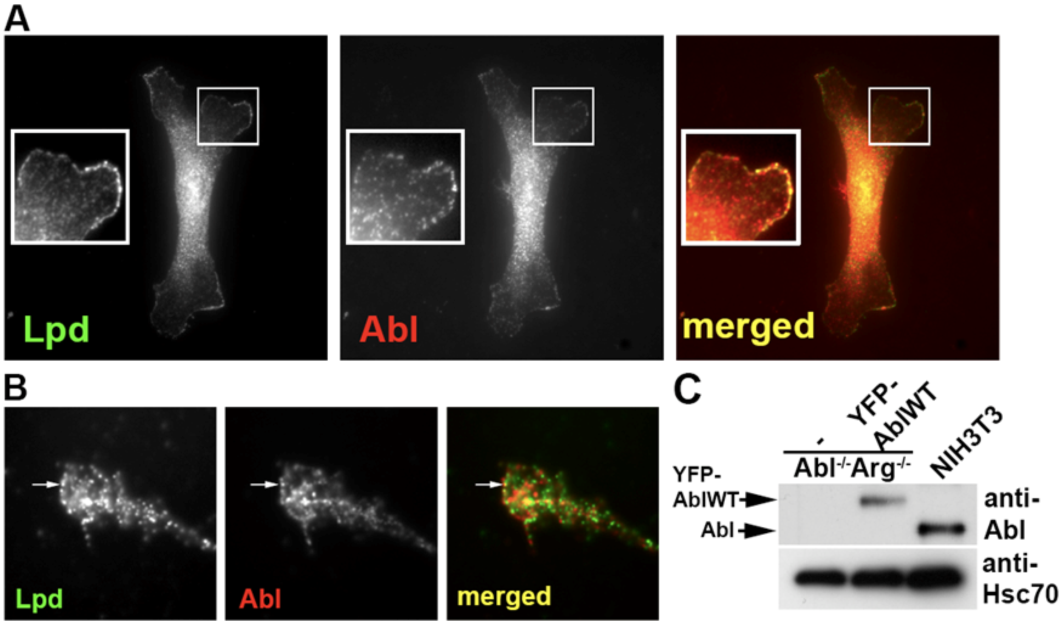

Figure S3.
Abl colocalises with Lpd at the leading edge in NIH3T3 fibroblasts and in primary hippocampal neurons. (A) NIH3T3 cells were plated on fibronectin and (B) primary hippocampal neurons on poly-D-lysine and fixed and stained with anti-Lpd and anti-c-Abl antibodies. Arrow points to an area of colocalization of Lpd and c-Abl at the leading edge.
(C) Analysis of Abl levels in $Abl^{-/-}Arg^{-/-}$, YFP-AblWT/$Abl^{-/-}Arg^{-/-}$ MEF's and NIH3T3 cells. $Abl^{-/-}Arg^{-/-}$ MEF's were reconstituted with wild type YFP-c-Abl and c-Abl levels were assessed in comparison to NIH3T3 fibroblast cells by Western blotting.

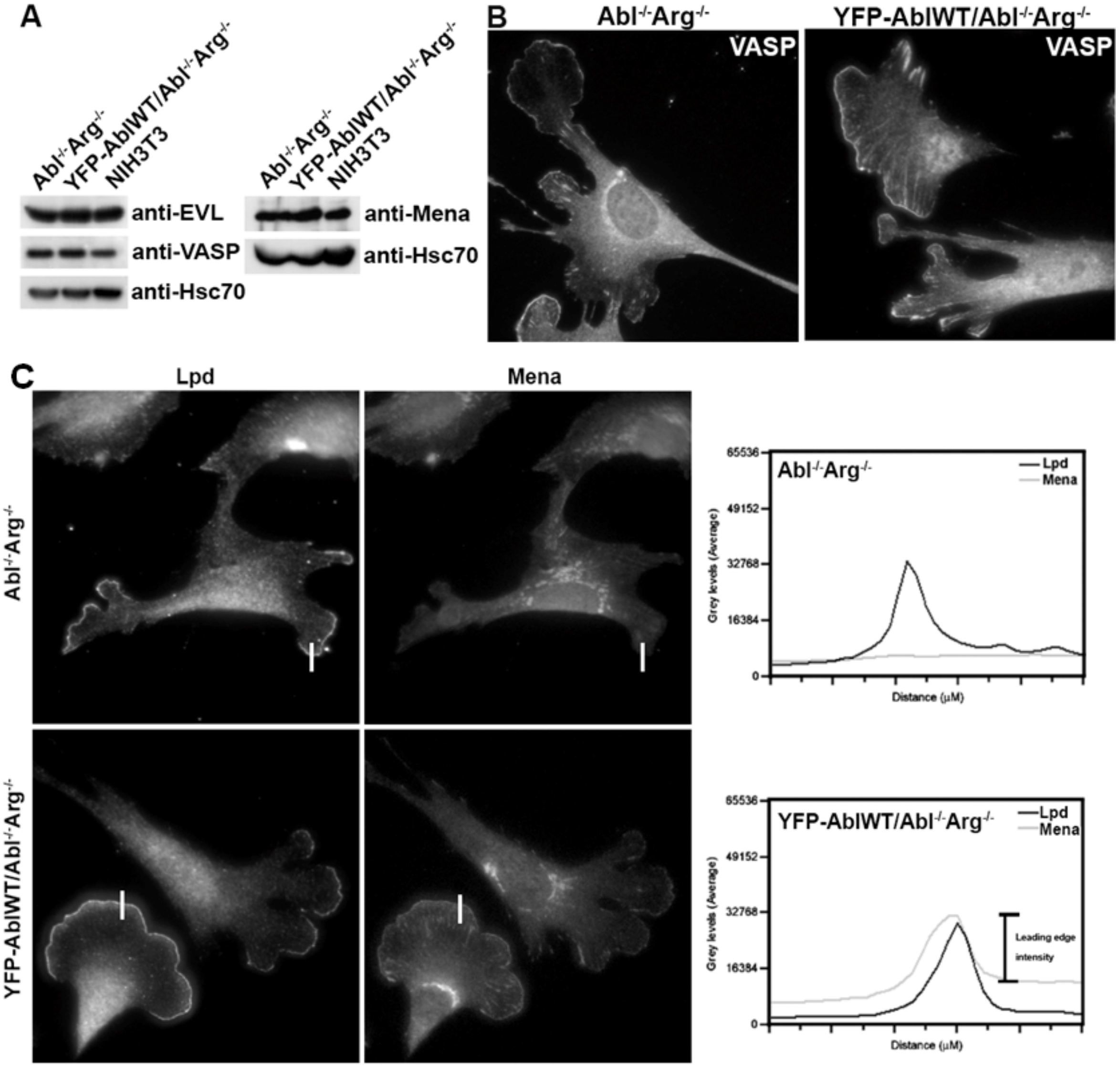

Figure S4.
Abl kinases regulate leading edge localisation of Mena and EVL but not VASP.
(A) Western blot analysis of lysates from Abl$^{-/-}$Arg$^{-/-}$, YFP-AblWT/Abl$^{-/-}$Arg$^{-/-}$, and NIH3T3 cells using EVL, VASP, and Mena antibodies.
(B) Abl$^{-/-}$Arg$^{-/-}$ and YFP-AblWT/Abl$^{-/-}$Arg$^{-/-}$ cells were plated, fixed and stained with VASP antibodies.
(C) Analysis of colocalisation of Lpd with Ena/VASP proteins at leading edge in both Abl$^{-/-}$Arg$^{-/-}$ and YFP-AblWT/Abl$^{-/-}$Arg$^{-/-}$ cells. Line scans were taken for Lpd and Mena immunofluorescence images of cells with Lpd positive leading edges. The leading edge intensity of Mena was calculated by taking the difference between the peak value and the background value of the Mena line scan. For each experiment, values above the median were scored as positive leading edge localisation and those below the median scored as cytosolic staining. Three independent experiments were performed and a minimum of 60 cells per cell line was quantified for each Ena/VASP protein.

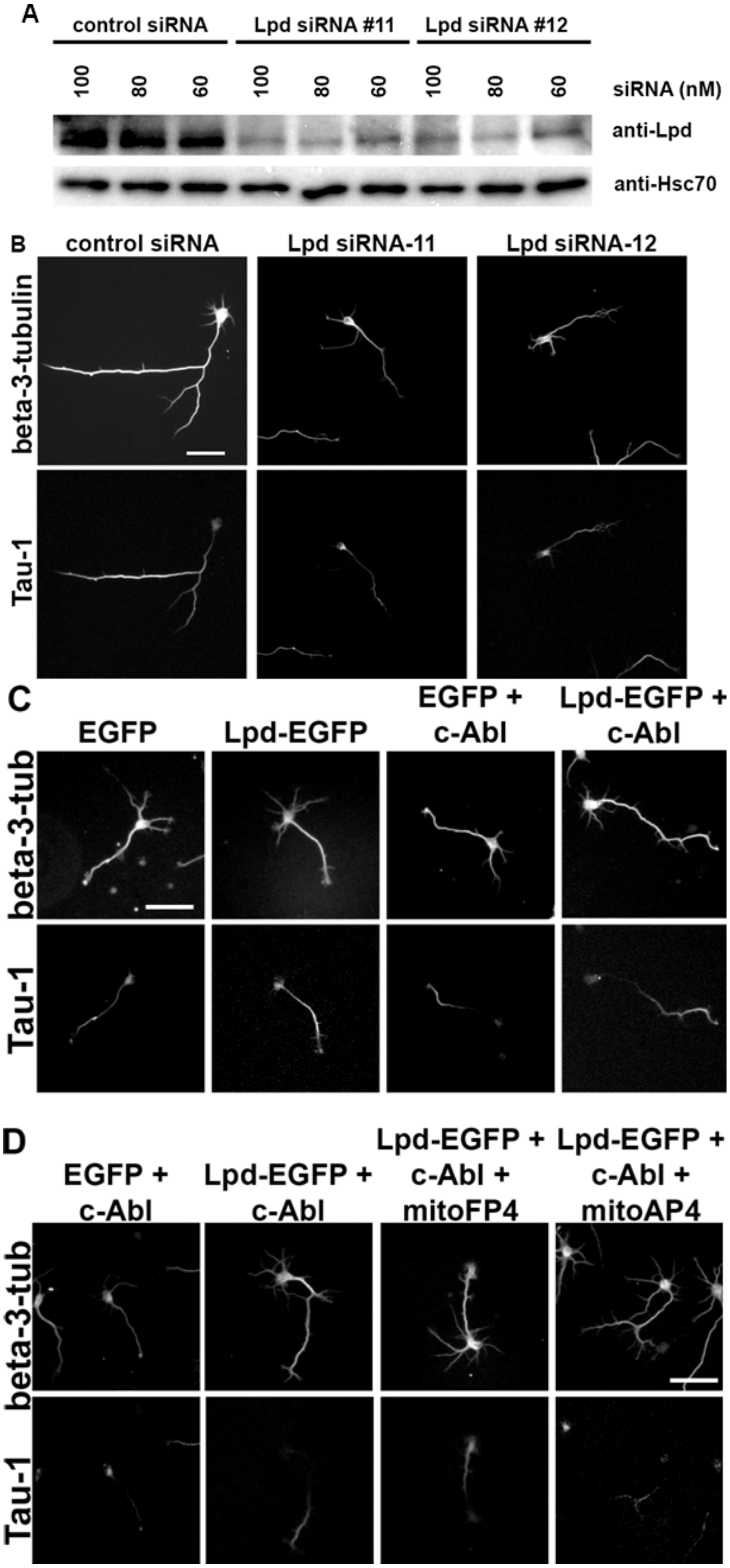
A
control siRNA
Lpd siRNA #11
Lpd siRNA #12
100
80
60
siRNA (nM)
anti-Lpd
anti-Hsc70
B
control siRNA
Lpd siRNA-11
Lpd siRNA-12
beta-3-tubulin
Tau-1
C
EGFP
Lpd-EGFP
EGFP + c-Abl
Lpd-EGFP + c-Abl
beta-3-tub
Tau-1
D
EGFP + c-Abl
Lpd-EGFP + c-Abl
Lpd-EGFP + c-Abl + mitoFP4
Lpd-EGFP + c-Abl + mitoAP4
beta-3-tub
Tau-1

Figure S5.

(A) Lpd expression can be knocked down by transfection of siRNA's. Scrambled control siRNA or two individual mouse Lpd specific siRNA's were transfected at different concentrations into B16F1 mouse melanoma cells and protein levels examined in a Western blot of cell lysates using anti-Lpd antibodies. Hsc70 antibodies served as a loading control.

(B) Primary hippocampal neurons transfected with Lpd specific siRNA's or non-targeting control siRNA plated on laminin coated coverslips were stained with anti-beta(III)-tubulin and Tau-1 antibodies.

(C) Primary hippocampal neurons transfected with EGFP vector control, Lpd-EGFP, wild type YFP-c-Abl+EGFP vector control, or Lpd-EGFP + wild type YFP-c-Abl plated on poly-D-lysine coated coverslips were stained with anti-beta(III)-tubulin and Tau-1 antibodies.

(D) Primary hippocampal neurons transfected with wild type YFP-c-Abl+EGFP vector control, or Lpd-EGFP + wild type YFP-c-Abl with mRFP1-FP4-mito or AP4-mito constructs and plated onto poly-D-lysine coated coverslips were stained with anti-beta(III)-tubulin and Tau-1 antibodies. Bar in (B,C,D): 50 μm.

Supplemental Experimental Procedures

Molecular Biology, Plasmids, and Reagents

pK1-c-Abl and Arg constructs [1, 2] VASP, Mena, EVL, and mRFP1-FP4/AP4-mito in pMSCV [3, 4] were kind gifts of Tony Koleske (Yale) and Frank Gertler (MIT), respectively. Lpd and RIAM [5, 6] were cloned into pENTR (Invitrogen), point mutations introduced using Quikchange® (Stratagene) and transferred into mammalian EGFP-, HA-, or GST-vectors using Gateway® recombination. SH2 and SH3 domain of c-Abl were cloned into pGEX-6P1 (Amersham) and GST-Abl-SH3 or -SH2 proteins were purified from E.coli on GSH-agarose. All constructs were verified by sequencing. Lpd ON-TARGETplus® siRNA's (J-043405-11, J-043405-12) and control siRNA No 2 (Dharmacon).

Antibodies: anti-Lpd pab 3917 [5], anti-VASP pab 2010 [3], anti-EVL mab 84H1 [7], anti-Mena mab A351F7D9 [8], anti-Hsc70 (Santa Cruz), anti-GST (Amersham), anti-Beta-III-tubulin pab (Covance), anti-Tau-1 mab (Chemicon), anti-Abl mab 8E9 (BD Biosciences), anti-phosphotyrosine 4G10 (Upstate), anti-p-Y426-Lpd and anti-p-Y1226-Lpd generated in rabbits with RASGI-p-YYVPKGK (421-432) SHISG-p-YATLRRG (1221-1232) and purified (Eurogentec). Secondary antibodies: HRP-goat anti-rabbit, goat anti-mouse, rabbit anti-goat (Dako).

Immunofluorescence Analysis and Imaging

For immunofluorescence analysis cells were plated on nitric acid washed coverslips (Hecht-Assistant) and fixed with 4% paraformaldehyde-PHEM (60mM PIPES, 25mM HEPES, 10mM EGTA, 2mM $MgCl_2$, 0.12M sucrose). Secondary antibodies: goat anti-rabbit, or anti-mouse Alexa488 or 568 (Molecular Probes) or goat-anti-mouse Cy5 (Zymed).

Primary hippocampal or cortical neurons, prepared from E16 mice as described for E18 rat [9], were transfected using the AMAXA Mouse Neuron Nucleofector kit and plated on poly-D-lysine or laminin (25 µg/ml) (BD) coated coverslips or dishes in Neurobasal, 5% FCS, 2% B27, 1% Glutamine, Pen/Strep and maintained without FCS. After 48 hr, cells were fixed (2% paraformaldehyde/PHEM), permeabilised (0.05% saponin, 10% FCS, TBS) and mounted (Prolong Gold, Invitrogen).

A Zeiss LSM510 or Olympus IX-81 microscope (Metamorph, Sutter filter wheels, ASI X-Y stage, Photometrics CascadeII 512B camera, 20xUPlanFL, 40xUPlanFL, 60xPlanApoNA1.45, or 100x UPlanApoS NA1.4 objectives) was used and dorsal ruffle formation analysed using a custom written journal for automated X-Y scanning of coverslips in Metamorph. Analysis of neuronal morphology was performed using NeuronJ [10].

In Vitro Kinase Assays

HEK293 cells were transfected with HA-Lpd and wild type c-Abl or wild type or dominant active c-Abl alone. c-Abl was immunoprecipitated (anti-c-Abl, K-12, Santa Cruz) from cell lysates and immunoprecipitate was incubated with purified GST or GST-Crk on glutathione

sepharose beads in the presence of γ-P^{32}-ATP. After extensive washes, beads were boiled with sample buffer and proteins resolved by SDS-PAGE. Phosphorylated GST-Crk in SDS-PAGE gel was detected using a phosphor-imager Typhoon 9200 (Amersham).

Supplemental References

1. Miller, A.L., Wang, Y., Mooseker, M.S., and Koleske, A.J. (2004). The Abl-related gene (Arg) requires its F-actin-microtubule cross-linking activity to regulate lamellipodial dynamics during fibroblast adhesion. J Cell Biol *165*, 407-419.
2. Moresco, E.M., Donaldson, S., Williamson, A., and Koleske, A.J. (2005). Integrin-mediated dendrite branch maintenance requires Abelson (Abl) family kinases. J Neurosci *25*, 6105-6118.
3. Bear, J.E., Loureiro, J.J., Libova, I., Fassler, R., Wehland, J., and Gertler, F.B. (2000). Negative regulation of fibroblast motility by Ena/VASP proteins. Cell *101*, 717-728.
4. Loureiro, J.J., Rubinson, D.A., Bear, J.E., Baltus, G.A., Kwiatkowski, A.V., and Gertler, F.B. (2002). Critical roles of phosphorylation and actin binding motifs, but not the central proline-rich region, for Ena/vasodilator-stimulated phosphoprotein (VASP) function during cell migration. Mol Biol Cell *13*, 2533-2546.
5. Krause, M., Leslie, J.D., Stewart, M., Lafuente, E.M., Valderrama, F., Jagannathan, R., Strasser, G.A., Rubinson, D.A., Liu, H., Way, M., et al. (2004). Lamellipodin, an Ena/VASP ligand, is implicated in the regulation of lamellipodial dynamics. Dev Cell *7*, 571-583.
6. Lafuente, E.M., van Puijenbroek, A.A., Krause, M., Carman, C.V., Freeman, G.J., Berezovskaya, A., Constantine, E., Springer, T.A., Gertler, F.B., and Boussiotis, V.A. (2004). RIAM, an Ena/VASP and Profilin ligand, interacts with Rap1-GTP and mediates Rap1-induced adhesion. Dev Cell *7*, 585-595.
7. Lanier, L.M., Gates, M.A., Witke, W., Menzies, A.S., Wehman, A.M., Macklis, J.D., Kwiatkowski, D., Soriano, P., and Gertler, F.B. (1999). Mena is required for neurulation and commissure formation. Neuron *22*, 313-325.
8. Lebrand, C., Dent, E.W., Strasser, G.A., Lanier, L.M., Krause, M., Svitkina, T.M., Borisy, G.G., and Gertler, F.B. (2004). Critical role of Ena/VASP proteins for filopodia formation in neurons and in function downstream of netrin-1. Neuron *42*, 37-49.
9. Goslin, K., and Banker, G. (1989). Experimental observations on the development of polarity by hippocampal neurons in culture. J Cell Biol *108*, 1507-1516.
10. Meijering, E., Jacob, M., Sarria, J.C., Steiner, P., Hirling, H., and Unser, M. (2004). Design and validation of a tool for neurite tracing and analysis in fluorescence microscopy images. Cytometry A *58*, 167-176.
